# The prevalence of Alzheimer disease in Odesa region

**DOI:** 10.1002/alz.088288

**Published:** 2025-01-09

**Authors:** Yurii Vorokhta, Iryna Khubetova

**Affiliations:** ^1^ Petro Mohyla Black Sea National University, Myckolayv, Myckolayv region Ukraine; ^2^ Medical House “ODREX”, Odesa, Odesa region Ukraine

## Abstract

**Background:**

The prevalence of dementia in people aged 65 and over in the world is approximately 6‐10%, two‐thirds of these cases are due to Alzheimer disease The frequency of deaths from Alzheimer disease in Ukraine is 0.3 per 100,000 population, while in Great Britain this indicator is 112 per 100,000 people, USA – 82, France – 76, Germany – 51 [1, 2].

The purpose of the study was to estimate the prevalence of Alzheimer’s disease in the Odesa region.

**Method:**

Retrospective analysis of the medical records about the registered cases of Alzheimer disease were received from Odesa Regional Mental Health Center for the years 2016‐2022. These data were analysed by the frequency and variance for indices of prevalence, age and gender structure.

**Result:**

According to the retrospective data of the Odesa Regional Mental Health Center for the years 2016‐2022, a constant increase in the number of detected patients with Alzheimer disease was noted ‐ from 4.9 cases per 100,000 population in 2016 to 6.3 cases in 2022. The gender balance throughout the analyzed period was stable, with a slight predominance (60%) of women in the structure of patients.

The average age of men was 72.8±0.3 years, women – 78.6±0.3 years. Statistically significant differences (p<0.05) could be explained by longer life expectancy in women.

**Conclusion:**

The prevalence of neurodegenerative diseases in the Odesa region, according to medical records, is significantly lower than the European average, which indicates underdiagnosis.

References:

1. 2023 Alzheimer’s disease facts and figures. Alzheimers Dement. 2023 Apr;19(4):1598‐1695. https://doi.org/10.1002/alz.13016. Epub 2023 Mar 14. PMID: 36918389.

2. Федотова М. С., Панфілова Г. Л., Цурікова О. В., Блажієвська О.М. Дослідження епідеміології деменції та хвороби Альцгеймера в Україні. [Research on the epidemiology of dementia and Alzheimer’s disease in Ukraine] Вісник фармації. 2021. 2(102) 50‐58 [Ukr]

